# Changes in Physical Fitness of Older People During the COVID-19 Pandemic in Germany: Objective Training Data

**DOI:** 10.1093/geroni/igab046.1772

**Published:** 2021-12-17

**Authors:** Jessica Koschate, Michel Hackbarth, Sandra Lau, Tania Zieschang

**Affiliations:** Carl von Ossietzky University Oldenburg, Oldenburg, Niedersachsen, Germany

## Abstract

The purpose of this study was to analyze objective training data on changes in leg muscle training before and after the COVID-19 lockdown during spring 2020 in Germany. Overall, the training data of 4435 individuals in the age group (AG) 45-64 years (55±5 years, 66% ♀) and of 2853 in the AG 65-95 years (72±6 years, 54% ♀) were exported from chip-controlled exercise circuits. Training weight and number of repetitions performed on the leg extensor were used to calculate a leg score (LS), considering the last three training sessions before the lockdown (baseline) and the first ten individual sessions as well as the averaged sessions for August, September and October after individual training resumption. Based on the baseline LS, three training intensity groups (TG_low, medium, high) were defined, and analyzed for differences (ANOVA). The LS in TG_low remained stable after the lockdown, but increased compared to baseline in both AGs after the first ten sessions (p<0.05). In TG_medium, LS was reduced at the first post training session (p<0.05) and returned to baseline levels at training session eight in the younger and session two in the older adults. In both AGs, LS was reduced in the TG_high (p<0.001), and did not reach baseline levels by October. Hence, the LS of TG_high was identified as being particularly affected by the training interruption, irrespective of age. More individually tailored training recommendations should be made for these individuals to be able to regain their initial training levels and avoid long-term adverse health effects.

